# Transmission Versus Truth, Imitation Versus Innovation: What Children Can Do That Large Language and Language-and-Vision Models Cannot (Yet)

**DOI:** 10.1177/17456916231201401

**Published:** 2023-10-26

**Authors:** Eunice Yiu, Eliza Kosoy, Alison Gopnik

**Affiliations:** Department of Psychology, University of California, Berkeley

**Keywords:** innovation, imitation, tool use, causal learning, children, large language models

## Abstract

Much discussion about large language models and language-and-vision models has focused on whether these models are intelligent agents. We present an alternative perspective. First, we argue that these artificial intelligence (AI) models are cultural technologies that enhance cultural transmission and are efficient and powerful imitation engines. Second, we explore what AI models can tell us about imitation and innovation by testing whether they can be used to discover new tools and novel causal structures and contrasting their responses with those of human children. Our work serves as a first step in determining which particular representations and competences, as well as which kinds of knowledge or skill, can be derived from particular learning techniques and data. In particular, we explore which kinds of cognitive capacities can be enabled by statistical analysis of large-scale linguistic data. Critically, our findings suggest that machines may need more than large-scale language and image data to allow the kinds of innovation that a small child can produce.

Recently, large language and language-and-vision models, such as OpenAI’s ChatGPT and DALL-E, have sparked much interest and discussion. These systems are trained on an unprecedentedly large amount of data and generate novel text or images in response to prompts. Typically, they are pretrained with a relatively simple objective such as predicting the next item in a string of text correctly. In some more recent systems, they are also fine-tuned both by their designers and through reinforcement learning by human feedback—humans judge the texts and images the systems generate and so further shape what the systems produce.

A common way of thinking about these systems is to treat them as individual agents and then debate how intelligent those agents are. The phrase “an AI” rather than “AI” or “AI system,” implying individual agency, is frequently used. Some have claimed that these models can tackle complex commands (e.g., Bubeck et al., 2023), perform abstract reasoning such as inferring theory of mind (e.g., Kosinski, 2023), and demonstrate creativity (e.g., Summers-Stay et al., 2023) in a way that parallels individual human agents.

We argue that this framing is wrong. Instead, we argue that the best way to think of these systems is as powerful new cultural technologies, analogous to earlier technologies such as writing, print, libraries, the Internet, and even language itself (Gopnik, 2022a, 2022b). Large language and vision models provide a new method for easy and effective access to the vast amount of text that others have written and images that others have shaped. These AI systems offer a new means for cultural production and evolution, allowing information to be passed efficiently from one group of people to another (Bolin, 2012; Boyd & Richerson, 1988; Henrich, 2018). They aggregate large amounts of information previously generated by human agents and extract patterns from that information.

This contrasts with perception and action systems that intervene on the external world and generate new information about it. This contrast extends beyond perception and action systems themselves. The kinds of causal representations that are embodied in theories, either scientific or intuitive, are also the result of truth-seeking epistemic processes (e.g., Gopnik & Wellman, 2012); they are evaluated with respect to an external world and make predictions about and shape actions in that world. New evidence from that world can radically revise them. Causal representations, like as perceptual representations, are designed to solve “the inverse problem” (Palmer, 1999): the problem of reconstructing the structure of a novel, changing, external world from the data that we receive from that world. Although such representations may be very abstract, as in scientific theories, they ultimately depend on perception and action—on being able to perceive the world and act on it in new ways.

These truth-seeking processes also underlie some AI systems. For example, reinforcement learning systems, particularly model-based systems, can be understood as systems that act on the world to solve something similar to an inverse problem. They accumulate data to construct models of the world that allow for broad and novel generalization. In robotics, in particular, systems such as these make contact with an external world, alter their models as a result, and allow for novel actions and generalizations, although these actions and generalizations are still very limited. Similarly, a number of AI approaches have integrated causal inference and theory formation into learning mechanisms in an attempt to design more human-like systems (Goyal & Bengio, 2022; Lake et al., 2015; Pearl, 2000). These systems are, however, very different from the typical large language and vision models that instead rely on relatively simple statistical inference applied to enormous amounts of existing data.

Truth-seeking epistemic processes contrast with the processes that allow faithful transmission of representations from one agent to another, regardless of the relation between those representations and the external world. Such transmission is crucial for abilities such as language learning and social coordination. There is considerable evidence that mechanisms for this kind of faithful transmission are in place early in development and play a particularly important role in human cognition and culture (Meltzoff & Moore, 1977; Meltzoff & Prinz, 2002).

However, such mechanisms may also be actively in tension, for good and ill, with the truth-seeking mechanisms of causal inference and theory formation. For example, in the phenomenon of “overimitation” human children (and adults) reproduce all the details of a complex action sequence even when they are not causally relevant to the outcome of that action (Lyons et al., 2011; Whiten et al., 2009).

Overimitation may increase the fidelity and efficiency of cultural transmission for complex actions. However, it also means that that transmission is not rooted in a causal understanding that could be altered by further evidence in a changing environment. Similarly, there is evidence that children begin by uncritically accepting testimony from others about the world and revise that testimony only when it is directly contradicted by other evidence (Harris & Koenig, 2006).

We argue that large language models (LLMs) enable and facilitate this kind of transmission in powerful and significant ways by summarizing and generalizing from existing text. However, nothing in their training or objective functions is designed to fulfill the epistemic functions of truth-seeking systems such as perception, causal inference, or theory formation. Even though state-of-the-art LLMs have been trained to estimate uncertainty over the validity of their claims (Kadavath et al., 2022), their output prediction probabilities do not distinguish between epistemic uncertainty, which relates to the lack of knowledge and can be resolved with more training data, and aleatoric uncertainty (relating to chance or stochasticity and so irreducible; Huang et al., 2023; Lin et al., 2023). The fact that such systems “hallucinate” (Azamfirei et al., 2023) is a well-known problem but badly posed—“hallucination” implies that the agent discriminates between veridical and nonveridical representations in the first place, and LLMs do not.

This contrast between transmission and truth is in turn closely related to the imitation/innovation contrast in discussions of cultural evolution in humans (Boyd & Richerson, 1988; Henrich, 2018; Legare & Nielsen, 2015; Tomasello et al., 1993). Cultural evolution depends on the balance between these two different kinds of cognitive mechanisms. Imitation allows the transmission of knowledge or skill from one person to another (Boyd et al., 2011; Henrich, 2016). Innovation produces novel knowledge or skill through contact with a changing world (Derex, 2022). Imitation means that each individual agent does not have to innovate—they can take advantage of the cognitive discoveries of others. But imitation by itself would be useless if some agents did not also have the capacity to innovate. It is the combination of the two that allows cultural and technological progress.

Of course, imitation and transmission may involve some kinds of generalization and novelty. A Wikipedia entry, for example, or even an old-fashioned newspaper article, is the result of multiple human editors collectively shaping new text that none of them could have generated alone. The result involves a kind of generalization and novelty. Large language models produce similar generalizations. Similarly, it may be possible at times to produce a kind of innovation simply by generalizing from actions that are already known. If I know that a 2-ft ladder will reach a shelf, I may be able to immediately infer that a taller ladder will allow me to reach an even higher shelf, even if I have not seen the ladder used that way before.

However, striking innovations that allow novel adaptations to novel problems and environments require inferences that go beyond the information that has already been acquired. These inferences may take off from existing causal models to generate new causal possibilities that are very different from those that have been observed or transmitted earlier, or they may inspire new explorations of the external world. From the AI perspective a useful way of thinking about it is that imitation involves a kind of interpolative generalization: Within what is already known, skills and knowledge are utilized, emulated, and shared across a variety of contexts. On the other hand, innovation reflects a more extrapolative or “out-of-distribution” generalization.

In any given case, it may be difficult to determine which kinds of cognitive mechanisms produced a particular kind of representation, behavior, knowledge, or skill. For example, my answer to an exam question in school might simply reflect the fact that I have remembered what I was taught, and I can make small generalizations from that teaching. Or it might indicate that I have knowledge that would allow me to make novel predictions about or perform novel actions on the external world. Probing the responses of large language models may give us a tool to help answer that question—at least, in principle. If large models that are trained only on language internal statistics can reproduce particular competencies, for example, producing grammatical text in response to a prompt, that suggests that those abilities can be developed through imitation—extracting existing knowledge encoded in the minds of others. If not, that suggests that these capacities may require innovation—extracting knowledge from the external world.

Thus, large language and vision models provide us with an opportunity to discover which representations and cognitive capacities, in general, human or artificial, can be acquired purely through cultural transmission itself and which require independent contact with the external world—a long-standing question in cognitive science (Barsalou, 2008; Gibson, 1979; Grand et al., 2022; Landauer & Dumais, 1997; Piantadosi, 2023).

In this article, we explore what state-of-the-art large language and language-and-vision models can contribute to our understanding of imitation and innovation. We contrast the performance of models trained on a large corpus of text data, or text and image data, with that of children.

## Large Language and Language-and-Vision Models as Imitation Engines

Imitation refers to the behavior of copying or reproducing features or strategies underlying a model’s behavior (Heyes, 2001; Tomasello, 1990). It implies interpolative generalization. The way the question or context is presented may vary, but the underlying behavior or idea stems from a repertoire of knowledge and skills that already exist. By observing and imitating others, individuals acquire the skills, knowledge, and conventions that are essential to effectively participate in their cultural groups, promoting cultural continuity over time. An assortment of technological innovations such as writing, print, the Internet, and—we would argue—LLMs, have made this imitation much more effective over time.

Moreover, cultural technologies not only allow access to information; they also codify, summarize, and organize that information in ways that enable and facilitate transmission. Language itself works by compressing information into a digital code. Writing and print similarly abstract and simplify from the richer information stream of spoken language while allowing at the same time wider temporal and spatial access to that information. Print, in addition, allows many people to receive the same information at the same time, and this is, of course, highly amplified by the Internet. At the same time, indexes, catalogs, libraries, and, more recently, Wikis and algorithmic search engines allow humans to quickly find relevant text and images and use those texts and images as a springboard to generate additional text and images.

Deep learning models trained on large data sets today excel at imitation in a way that far outstrips earlier technologies and so represent a new phase in the history of cultural technologies. Large language models such as Anthropic’s Claude and OpenAI’s ChatGPT can use the statistical patterns in the text in their training sets to generate a variety of new text, from emails and essays to computer programs and songs. GPT-3 can imitate both natural human language patterns and particular styles of writing close to perfectly. It arguably does this better than many people (M. Zhang & Li, 2021). Strikingly and surprisingly, the syntactic structure of the language produced by these systems is accurate. There is some evidence that large language models can even grasp language in more abstract ways than humans and imitate human figurative language understanding (e.g., Jeretic et al., 2020; Stowe et al., 2022). This suggests that finding patterns in large amounts of human text may be enough to pick up many features of language, independent of any knowledge about the external world.

In turn, this raises the possibility that children learn features of language or images in a similar way. In particular, this discovery has interesting connections to the large body of empirical literature showing that infants are sensitive to the statistical structure of linguistic strings and visual images from a very young age (e.g., Kirkham et al., 2002; Saffran et al., 1996). The LLMs suggest that this may enable much more powerful kinds of learning than we might have thought, such as the ability to learn complex syntax.

On the other hand, although these systems allow skilled imitation, the imitation that they facilitate may differ from that of children in important ways. There are debates in the developmental literature about how much childhood imitation simply reflects faithful cultural transmission (as in the phenomenon of overimitation) and how much it is shaped by and in the service of broader truth-seeking processes such as understanding the goals and intentions of others. Children can meaningfully decompose observed visual and motor patterns in relation to the agent, target object, movement path, and other salient features of events (Bekkering et al., 2000; Gergely et al., 2002). Moreover, children distinctively copy intentional actions (Meltzoff, 1995), discarding apparently failed attempts, mistakes, and causally inefficient actions (Buchsbaum et al., 2011; Schulz et al., 2008) when they seek to learn skills from observing other people (Over & Carpenter, 2013). Although the imitative behavior of large language and vision models can be viewed as the abstract mapping of one pattern to another, human imitation appears to be mediated by goal representation and the understanding of causal structure from a young age. It would be interesting to see whether large models also replicate these features of human imitation.

## Can Large Language and Language-and-Vision Models Innovate?

### Can LLMs discover new tools?

Where might we find empirical evidence for this contrast between transmission and truth, imitation and innovation? One important and relevant set of capacities involves tool use and innovation. The most ancient representative of the human genus is called *Homo habilis* (“handy man”) because of their ability to discover and use novel stone tools. Tool use is one of the best examples of the advantages of cultural transmission and of the balance between imitation and innovation. Imitation allows a novice to observe a model and reproduce their actions to bring about a particular outcome, even without understanding entirely the fine physical mechanisms and causal properties of the tool. Techniques such as “behavior cloning” in AI and robotics use a similar approach.

Again, however, the ability to imitate and use existing tools in an interpolative way depends on the parallel ability to discover new tools in an extrapolative way. Tool innovation is an indispensable part of human lives, and it has also been observed in a variety of nonhuman animals such as crows (Von Bayern et al., 2009) and chimpanzees (Whiten et al., 2005). Tool innovation has often been taken to be a distinctive mark of intelligence in biological systems (Emery & Clayton, 2004; Reader & Laland, 2002).

Tool use can then be a particularly interesting point of comparison for understanding imitation and innovation in both models and children. Both computational models and humans can encode information about objects (e.g., Allen et al., 2020), but their capabilities for tool imitation versus tool innovation might differ. In particular, our hypothesis would predict that the models might capture familiar tool uses well (e.g., predicting appropriately that a hammer should be used to bang in a nail). However, these systems might have more difficulty producing the right responses for tool innovation involving unusual or novel tools, which depends on discovering and using new causal properties, functional analogies, and affordances.

We might, however, also wonder whether young children can themselves perform this kind of innovation, or whether it depends on explicit instruction and experience. Physically building a new tool from scratch and then executing a series of actions that lead to a desired goal is a difficult task for young children (Beck et al., 2011). But children might find it easier to recognize new functions in everyday objects and to select appropriate object substitutes in the absence of typical tools to solve various physical tasks. In an ongoing study of tool innovation (Yiu & Gopnik, 2023), we have investigated whether human children and adults can insightfully use familiar objects in new ways to accomplish particular outcomes and compared the results to the output of large deep learning models such as GPT-3 and GPT-4.

Tool innovation can involve designing new tools from scratch, but it can also refer to discovering and using old tools in new ways to solve novel problems (Rawlings & Legare, 2021). We might think of this as the ability to make an out-of-distribution generalization about a functional goal. Our experiment examines the latter type of tool innovation.

Our study has two components: an “imitation” component (making an interpolative judgment from existing knowledge about objects) and an “innovation” component (making an extrapolative judgment about the new ways that objects could be used). In the innovation part of the study, we present a series of problems in which a goal has to be executed in the absence of the typical tool (e.g., drawing a circle in the absence of a compass). We then provide alternative objects for participants to select: (a) an object that is more superficially similar to the typical tool and is associated with it but is not functionally relevant to the context (e.g., a ruler), (b) an object that is superficially dissimilar but has the same affordances and causal properties as the typical tool (e.g., a teapot that possesses a round bottom), and (c) a totally irrelevant object (e.g., a stove). In the imitation part of the study, we present the same sets of objects but ask participants to select which of the object options would “go best” with the typical tool (e.g., a compass and a ruler are more closely associated than a compass and a teapot).

So far, we have found that both children aged 3 to 7 years old presented with animations of the scenario (*n* = 42, *M*_age_ = 5.71 years, *SD* = 1.24) and adults (*n* = 30, *M*_age_ = 27.80 years, *SD* = 5.54) can recognize common superficial relationships between objects when they are asked which objects should go together (*M*_children_ = 88.4%, *SE*_children_ = 2.82%; *M*_adults_ = 84.9%, *SE*_adults_ = 3.07%). But they can also discover new functions in everyday objects to solve novel physical problems and so select the superficially unrelated but functionally relevant object (*M*_children_ = 85.2%, *SE*_children_ = 3.17%; *M*_adults_ = 95.7%, *SE*_adults_ = 1.04%). In ongoing work, we have found that children demonstrate these capacities even when they receive only a text description of the objects, with no images.

Using exactly the same text input that we used to test our human participants, we queried OpenAI’s GPT-4, gpt-3.5-turbo, and text-davinci-003 models; Anthropic’s Claude; and Google’s FLAN-T5 (XXL). Because we noticed that the models could alter their responses depending on how the order of options was presented, we queried the models six times for every scenario to account for the six different orders that could be generated by the three options. We set model outputs as deterministic with a temperature of 0 and kept the default values for all other parameters (Binz & Schulz, 2023; Hu et al., 2022). We averaged the scores (1 for selecting the relevant object and 0 for any other response) across the six repeated trials. As we predicted we found that these large language models are almost as capable of identifying superficial commonalities between objects as humans are. They are sensitive to the superficial associations between the objects, and they excel at our imitation tasks (*M*_GPT4_ = 83.3%, *SE*_GPT4_ = 4.42%; *M*_gpt-3.5-turbo_ = 73.1%, *SE*_gpt-3.5-turbo_ = 5.26%; *M*_davinci_ = 59.9%, *SE*_davinci_ = 5.75%; *M*_Claude_ = 69.9%, *SE*_Claude_ = 5.75%; *M*_Flan_ = 74.8%, *SE*_Flan_ = 5.17%)—they generally respond that the ruler goes with the compass. However, they are less capable than humans when they are asked to select a novel functional tool to solve a problem (*M*_GPT4_ = 75.9%, *SE*_GPT4_ = 4.27%; *M*_gpt-3.5-turbo_ = 58.9%, *SE*_gpt-3.5-turbo_ = 5.64%; *M*_davinci_ = 8.87%, *SE*_davinci_ = 2.26%; *M*_Claude_ = 58.16%, *SE*_Claude_ = 6.06%; *M*_Flan_ = 45.7%, *SE*_Flan_ = 5.42%)—they again choose the ruler rather than the teapot to draw a circle. This suggests that simply learning from large amounts of existing language may not be sufficient to achieve tool innovation. Discovering novel functions in everyday tools is not about finding the statistically nearest neighbor from lexical co-occurrence patterns. Rather, it is about appreciating the more abstract functional analogies and causal relationships between objects that do not necessarily belong to the same category or are associated in text. In these examples, people must use broader causal knowledge, such as understanding that tracing an object will produce a pattern that matches the object’s shape, to produce a novel action that has not been observed or described before, in much the same way a scientific theory, for example, allows novel interventions on the world (Pearl, 2000). Compared with humans, large language models are not as successful at this type of innovation task. On the other hand, they excel at generating responses that simply demand some abstraction from existing knowledge.

One might ask whether success on our task also requires visual and spatial information rather than merely text. Indeed, GPT-4, a large multimodal model that is trained on larger amounts of images and text, demonstrates better performance than the other large language models on both the innovative and imitative tasks. Nevertheless, despite the massive amounts of vision and language training data, it is still not as innovative as human adults are when they discover new functions in existing objects. It is also unclear whether GPT-4’s improved performance stems from its multimodal character or from reinforcement learning from human feedback—a point we return to later.

### Can LLMs discover novel causal relationships and use them to design interventions?

Discovering novel tools depends on being able to infer a novel causal relationship, such as drawing a circle by tracing the bottom of a teapot. A substantial amount of research shows that even very young children excel at discovering such relationships. Information about causal structure can be conveyed through imitation and cultural transmission. In fact, from a very young age, even infants will reproduce an action they have observed to bring about an effect (Waismeyer et al., 2015). However, very young children can also infer novel causal structure by observing complex statistical relations among events, and most significantly, by acting on the world themselves to bring about effects like a scientist performing experiments (Cook et al., 2011; Gopnik et al., 2004, 2017; Gopnik & Tenenbaum, 2007; Schulz et al., 2007). Causal discovery is a particularly good example of a cognitive process that is directed at solving an inverse problem and discovering new truths through perception and action. Moreover, these processes of causal discovery do not depend on particular assumptions about “intuitive physics.” Very young children can make such inferences about psychological and social relationships as well as physical ones, and they can discover new causal relations that actually contradict the assumptions of intuitive physics (Gopnik & Wellman, 2012).

In another line of research (Kosoy et al., 2022, 2023), we have explored whether LLMs and other AI models can discover and use novel causal structure. In these studies we use a virtual “blicket detector”—a machine that lights up and plays music when you put some objects on it but not others. The blicket detector can work on different abstract principles or “overhypotheses”: individual blocks may activate it, or you may need a combination of blocks to do so. An overhypothesis refers to an abstract principle that reduces a hypothesis space at a less abstract level (Kemp et al., 2007), and a causal overhypothesis refers to transferable abstract hypotheses about sets of causal relationships (Kosoy et al., 2022). If you know that it takes two blocks to make the machine go, you will generate different specific hypotheses about which blocks are blickets.

The blicket detector tasks intentionally involve a new artifact, described with new words, so that the participants cannot easily use past culturally transmitted information, such as the fact that flicking a light switch makes a bulb go on. Assumptions about intuitive physics will also not enable a solution. In these experiments, we simply ask children to figure out how the machines work and allow them to freely explore and act to solve the task and determine which blocks are blickets. Even 4-year-old children spontaneously acted on the systems and discovered their structure—they figured out which ones were blickets and used them to make the machine go.

We then gave a variety of LLMs, including OpenAI’s ChatGPT, Google’s PaLM, and most recently LaMDA, the same data that the children produced, described in language (e.g., “I put the blue one and the red one on the machine and the machine lit up”) and prompted the systems to answer questions about the causal structure of the machine (e.g., “Is the red one a blicket?”).

LLMs did not produce the correct causal overhypotheses from the data. Young children, in contrast, learned novel causal overhypotheses from only a handful of observations, including the outcome of their own experimental interventions, and applied the learned structure to novel situations. In contrast, large language models and vision-and-language models, as well as both deep reinforcement learning algorithms and behavior cloning, struggled to produce the relevant causal structures, even after massive amounts of training compared with children. This is consistent with other recent studies: LLMs produce the correct text in cases such as causal vignettes, in which the patterns are available in the training data, but often fail when they are asked to make inferences that involve novel events or relations in human thought (e.g., Binz & Schulz, 2023; Mahowald et al., 2023), sometimes even when these involve superficially slight changes to the training data (e.g., Ullman, 2023).

## Challenges of Studying Large Language and Language-and-Vision Models: The Questions Left Unanswered

It is difficult to escape the language of individual agency, for example, to ask whether AI can or cannot innovate (e.g., González-Díaz & Palacios-Huerta, 2022; Stevenson et al., 2022), solve a causal problem (e.g., Kıcıman et al., 2023), or even can or cannot be sentient or intelligent (e.g., Mitchell, 2023). A great deal of the discussion about AI has this character. But we emphasize again that the point of this work is neither to decide whether or not LLMs are intelligent agents nor to present some crucial comparative “gotcha” test that would determine the answer to such questions in AI systems. Instead, the research projects we have briefly described here are a first step in determining which representations and competences, as well as which kinds of knowledge or skill, can be derived from which learning techniques and data. Which kinds of knowledge can be extracted from large bodies of text and images, and which depend on actively seeking the truth about an external world?

We want to emphasize again that other AI systems, such as model-based reinforcement learning or causal inference systems, may indeed more closely approximate truth-seeking cognitive systems. In fact, we also evaluated the performance of other AI systems, including two popular deep reinforcement learning algorithms, Advantage Actor Critic (A2C) and Proximal Policy Optimization Version 2 (PPO2), which are trained on all possible overhypotheses prior to the test trials. Although these systems are conceptually closer to the truth-seeking systems children use, they are still extremely limited in comparison.

There is a great deal of scope for research that uses developmental-psychology techniques to investigate AI systems and vice versa (Frank, 2023). Of course, there are anecdotal instances in which large language models seem to exhibit intelligent-like behaviors (Bubeck et al., 2023), solving arguably novel and complex tasks from physics and mathematics to story writing and image generation. Nonetheless, developmentalists have long realized that superficially similar behaviors, including creative- and innovative-like behaviors in humans and AI models, can have very different psychological origins and can be the result of very different learning techniques and data. As a result, we have put considerable methodological energy into trying to solve this problem. A particular conversation with a child, however compelling, is just the start of a proper research program including novel, carefully controlled tests such as our tests of tool innovation and novel causal inference. The conversation may reflect knowledge that has come through imitation within the training data set, statistical pattern recognition, reinforcement from adults, or conceptual understanding; the job of the developmental psychologist is to distinguish these possibilities. This should also be true of our assessments of AI systems.

At the same time AI systems have their own properties that need to be considered when we compare their output to that of humans. These can sometimes be problematic; for example, once a particular cognitive test is explicitly described in Internet text, it then becomes part of a large language model’s training sample—we found that in some cases the systems referred to our earlier published blicket-detector articles as the source for their answers. There are many more cases in the literature in which large language models, including GPT-4, seem to respond to novel examples sampled from the training distribution almost perfectly (again reinforcing that they are perfect imitators) but then fail miserably to generalize to out-of-distribution examples that require the discovery of more abstract causal hypotheses and innovation (e.g., Chowdhery et al., 2022; Talmor et al., 2020; H. Zhang et al., 2022).

In addition, the more recent versions of GPT, GPT-4, and GPT-3.5, have also been fine-tuned through reinforcement learning from human feedback. This also raises problems. Reinforcement learning from human feedback may itself be considered a method for enabling cultural transmission. However, in practice, we know very little about exactly what kinds of feedback these systems receive or how they are shaped by that feedback. Reinforcement learning from human feedback is both opaque and variable and may simply edit out the most obvious mistakes and errors.

On the other hand, these systems, particularly the “classic” LLMs, also have the advantage that we know more about their data and learning techniques than we do about those of human children. For example, we know that the data for GPT systems is Internet text and that the training function involves predicting new text from earlier text. We know that large language models and language-and-vision models are built on deep neural networks and trained on immense amounts of unlabeled text or text-image pairings.

These kinds of techniques may indeed contribute to some kinds of human learning as well. Children do learn through cultural transmission and statistical generalizations from data. But human children also learn in very different ways. Although we do not know the details of children’s learning algorithms or data, we do know that, unlike large language and language-and-vision models, children are curious, active, self-supervised, and intrinsically motivated. They are capable of extracting novel and abstract structures from the environment beyond statistical patterns, spontaneously making overhypotheses and generalizations, and applying these insights to new situations.

Because performance in large deep learning models has been steadily improving with increasing model size on various tasks, some have advocated that simply scaling up language models could allow task-agnostic, few-shot performance (e.g., Brown et al., 2020). But a child does not interact with the world better by increasing their brain capacity. Is building the tallest tower the ultimate way to reach the moon? Putting scale aside, what are the mechanisms that allow humans to be effective and creative learners? What in a child’s “training data” and learning capacities is critically effective and different from that of LLMs? Can we design new AI systems that use active, self-motivated exploration of the real external world as children do? And what we might expect the capacities of such systems to be? Comparing these systems in a detailed and rigorous way can provide important new insights about both natural intelligence and AI.

## Conclusion

Large language models such as ChatGPT are valuable cultural technologies. They can imitate millions of human writers, summarize long texts, translate between languages, answer questions, and code programs. Imitative learning is critical for promoting and preserving knowledge, artifacts, and practices faithfully within social groups. Moreover, changes in cultural technologies can have transformative effects on human societies and cultures—for good or ill. There is a good argument that the initial development of printing technology contributed to the Protestant Reformation. Later improvements in printing technology in the 18th century were responsible for both the best parts of the American Revolution and the worst parts of the French Revolution (Darnton, 1982). Large language and language-and-vision models may well have equally transformative effects in the 21st century.

However, cultural evolution depends on a fine balance between imitation and innovation. There would be no progress without innovation—the ability to expand, create, change, abandon, evaluate, and improve on existing knowledge and skills. Whether this means recasting existing knowledge in new ways or creating something entirely original, innovation challenges the status quo and questions the conventional wisdom that is the training corpus for AI systems. Large language models can help us acquire information that is already known more efficiently, even though they are not innovators themselves. Moreover, accessing existing knowledge much more effectively can stimulate more innovation among humans and perhaps the development of more advanced AI. But ultimately, machines may need more than large-scale language and images to match the achievements of every human child.

## Transparency

*Action Editor:* Mirta Galesic

*Editor:* Interim Editorial Panel
